# A review: Marine aquaculture impacts marine microbial communities

**DOI:** 10.3934/microbiol.2024012

**Published:** 2024-03-19

**Authors:** Xiao Zhang, Jia Hua, Zule Song, Kejun Li

**Affiliations:** College of Marine Ecology and Environment, Shanghai Ocean University, Shanghai, China

**Keywords:** mariculture, microbial community, microbial diversity, antibiotic resistance genes (ARGs), integrated multi-trophic aquaculture (IMTA)

## Abstract

Marine aquaculture is key for protein production but disrupts marine ecosystems by releasing excess feed and pharmaceuticals, thus affecting marine microbes. Though vital, its environmental impact often remains overlooked. This article delves into mariculture's effects on marine microbes, including bacteria, fungi, viruses, and antibiotic-resistance genes in seawater and sediments. It highlights how different mariculture practices—open, pond, and cage culture—affect these microbial communities. Mariculture's release of nutrients, antibiotics, and heavy metals alters the microbial composition, diversity, and functions. Integrated multi-trophic aquaculture, a promising sustainable approach, is still developing and needs refinement. A deep understanding of mariculture's impact on microbial ecosystems is crucial to minimize pollution and foster sustainable practices, paving the way for the industry's sustainable advancement.

## 1. Introduction

The vast expanse of the ocean, which is the largest habitat on earth, harbors a diverse array of microorganisms pivotal to drive productivity. The distinctive marine environment profoundly shapes microbial diversity, with observed declines in both bacterial and viral diversity corresponding to an increased seawater depth [1]. Microorganisms play a pivotal role in global biogeochemical cycles. Human activities introduce nutrients into the ocean through direct discharges, rivers, and underground runoff. This anthropogenic influence alters the community composition of waterborne microorganisms, thus fostering increased species diversity and consequential impacts on community functions [2]. Moreover, sewage discharge may introduce human pathogenic bacteria, posing potential threats to human health.

Mariculture plays a crucial role in bolstering global food security by providing affordable aquatic food, particularly in malnourished regions [3]. Its efficient production cycles and high yield per unit area enable the cultivation of economically valuable organisms such as fish, shellfish, and shrimp. These marine organisms contribute essential biological components, including polysaccharides, polypeptides, and long-chain bioactive substances vital for human cardiovascular health [4]–[7]. With the global population on the rise, traditional farming methods struggle to meet the growing demand for seafood. Mariculture emerges as a promising solution to alleviate the pressure on wild aquatic organisms, thus ensuring ecosystem stability while satisfying the increasing demand for fishery resources [8].

The expansion of coastal aquaculture, particularly through intensive practices in seawater, has significantly boosted seafood production [9]. Marine aquaculture capitalizes on the rich protein, vitamins, and micronutrients present in fishery resources, with a focus on economically valuable species such as fish, shellfish, and algae [10]. In the open ocean, there are abundant opportunities for fish growth, and mariculture plays a pivotal role in ensuring a sustainable food fish supply. Despite challenges posed by ocean currents and seawater temperature, strategic measures, including selective breeding methods such as sexual breeding, are employed to mitigate production losses [11]. Cultivating mollusks and algae contributes to environmental sustainability by absorbing organic waste and nutrients, effectively reducing eutrophication, and minimizing adverse impacts on the ecological environment [12].

High-density rafts and cages hinder seawater circulation, adversely affecting marine life growth, compromising the ocean's self-healing capacity, and causing ecological and environmental issues [12]. With an increased awareness of environmental pollution, the conventional monoculture model in marine aquaculture proves inadequate to evolve development needs. To achieve sustainable farming, the integrated multi-trophic aquaculture (IMTA) gains prominence in coastal waters. IMTA strategically combines the aquacultures of fish, shrimp, shellfish, sea cucumbers, sea urchins, algae, and other organisms with diverse nutritional levels. This approach leverages waste from fish and shrimp breeding to benefit shellfish and sea urchins, while algae and aquatic plants efficiently utilize inorganic substances from aquatic animal breeding [13].

Research indicates that bioactive substances from diverse microorganisms exhibit complex structures and diverse biological activities, including antibacterial and anti-tumor properties, thus highlighting significant developmental potential [14]. Mariculture has been shown to impact the microbial community composition and diversity, thereby disrupting the biosynthesis, transport, and metabolism of microorganisms in the water, leading to increased phylogenetic similarities among specific microbes [15],[16]. A comprehensive understanding of the impact of marine aquaculture on microbial communities is crucial for sustainable development and addressing existing challenges. This article systematically reviews how mariculture influences the structure and diversity of microbial communities, thereby encompassing bacteria, fungi, and viruses in seawater and sediments, as well as its effects on the distribution of antibiotic resistance genes (ARGs), thus forecasting that IMTA systems appear promising for addressing various challenges in mariculture.

## 2. The impact of mariculture on marine bacterioplankton communities

### 2.1. Effects of open marine culture on planktonic microorganisms

In open marine culture, bacterioplankton predominantly consists of Proteobacteria, Actinobacteria, Bacteroidetes, and Cyanobacteria [17],[18]. In the breeding waters of *Pyropia haitanensis* (Rhodophyta), bacterial communities become more diverse and abundant than in non-breeding waters, showcasing a significant presence of Rhodobacteraceae and Flavobacteriaceae [19]. During the overwintering period of *Larimichthys crocea* (Chordata, Actinopterygii), the bacterioplankton mainly performs chemo-heterotrophy and aerobic chemo-heterotrophy, with seasonal shifts in both abundance and function. Autumn sees a predominance of either animal parasitism or symbiosis, notably with *Acinetobacter baumannii* and *Moraxella* (Gammaproteobacteria), while spring shifts focus towards organic matter degradation, with *Rhodococcus* (Actinobacteria) taking a leading role [18]. At the artificial fishery habitat in the Pearl River estuary (China), bacterioplankton communities' alpha diversities peak in summer, as influenced by seasonal changes. These communities' metabolic activities, such as amino acid transport and metabolism, along with coenzyme transport and metabolism, increase oxygen consumption, which underscores dissolved oxygen's key role in community dynamics [20]. Laoshan Bay in the Yellow Sea experiences fluctuations in bacterioplankton diversity, decreasing from spring to summer and rebounding from autumn to winter, with the lowest abundance and highest diversity occurring in March [21]. Moreover, seasonal variations affect gene expression, with environmental information processing genes dominating in May, metabolism-related genes in November, and genes related to cellular processes and genetic information processing in March [21].

### 2.2. Effects of seawater pond culture on planktonic microorganisms

Long-term intensive seawater aquaculture induces seasonal variations in bacterioplankton composition, with prevalent bacteria such as Proteobacteria, Bacteroidetes, Firmicutes, and Actinobacteria [22]. In shrimp marine culture, water eutrophication induces changes in the bacterioplankton composition and diversity. Increased eutrophication leads to a heightened diversity and unpredictability in the bacterial community composition due to nutrient enrichment [23]. Intensive shrimp cultivation of *Litopenaeus vannamei* (Arthropoda, Malacostraca) impacts the bacterioplankton communities in culture water, as characterized by an initial increase in the Proteobacteria and Bacteroidetes levels, dominance of Cyanobacteria and Rhodobacteraceae in the early stages, followed by a decline, and an increase in Actinobacteria and Microbacteriaceae in later stages [24]. Shrimp health during breeding influences bacterioplankton, with healthy ponds exhibiting more Flavobacteriaceae and Actinobacteria, while diseased ponds show more Alphaproteobacteria and Gammaproteobacteria [25]. The functions of bacterioplankton in shrimp ponds remain stable, with differentially expressed genes associated with metabolism, genetic information processing, and environmental information processing. Additional functions include membrane transport, amino acid metabolism, and carbohydrate metabolism [26]. Bacterioplankton isolates from shrimp ponds display biosynthetic potential, with gene clusters related to terpene metabolism, polyketide synthases, and post-translationally modified peptides [27].

Seaweed functions as a substratum facilitating microbial attachment and the resulting organic matter contributes to bacterial reproduction and biofilm formation [28]. Oxygen production and polysaccharide secretion during seaweed cultivation are energy sources for bacterial growth [29]–[31]. Additionally, seaweed serves as a habitat for bacterial communities, providing resilience against predation or environmental fluctuations and fostering community survival [32],[33]. Algal cultivation influences the composition and alpha diversity of bacterial communities. A study in the cultivation areas of *Gracilariopsis lemaneiformis* (Rhodophyta) and adjacent uncultured areas near Nan'ao Island in the South China Sea demonstrated an improved water quality, the presence of degradable substances in the surface water, and an increased potential microbial abundance with the heightened alpha diversity of bacterial communities, notably associated with seaweed agar [34].

### 2.3. Effects of sea cage culture on planktonic microorganisms

Central to marine aquaculture, shellfish primarily feed on algae and organic debris [35]. Marine bacteria, which are widespread in shellfish culture waters, actively participate in material circulation and energy flow processes, thus influencing the bacterial community in seawater through shellfish cultivation [36]. During the cultivation of *Chlamys farreri* (Mollusca, Bivalvia), an increase in the concentration of PO_4_^3−^ in phosphorus-deficient waters led to a rise in the abundance of heterotrophic bacteria, *Synechococcus* (Cyanobacteria), and *Prochlorococcus*, among other bacteria [37]. Shellfish farming induces changes in the alpha diversity of seawater bacteria, with *Mytilus edulis* (Mollusca, Bivalvia) culture areas revealing involvement in functional pathways related to the single cycle and degradation of the aromatic compound gallate. The *M. edulis* culture increases the alpha diversity and the Simpson index [38]–[40]. The influence of shellfish culture on bacterioplankton diversity varies seasonally. Observations in scallop breeding areas along the Bohai Sea reveal marked seasonal variations in Proteobacteria, Cyanobacteria, Actinobacteria, and Bacteroidetes, with higher bacterial abundance near scallop breeding farms during summer compared to autumn [41].

Mariculture-induced environmental changes result in discernible functional differences in bacterial communities in water bodies. Near salmon cage farming, the bacterioplankton community predominantly exhibits functional gene annotations related to amino acid, nucleotide, and vitamin biosynthesis, potentially influenced by fish farming practices [42]. Furthermore, the presence of antibiotic resistance pathways near breeding farms suggests a potential correlation with antibiotic use during breeding [42]. In shrimp breeding areas, the analysis of functional gene annotations indicates an increased abundance in pathways associated with amino acid and carbohydrate metabolism [43].

As primary producers in the marine ecosystem, algae contribute through photosynthesis by generating dissolved oxygen and organic matter, thus simultaneously promoting the vitality of beneficial bacteria and inhibiting the proliferation of pathogenic bacteria in marine aquaculture [44],[45]. Distinct seaweed farming areas create diverse environmental conditions, thus influencing the composition of bacterial communities. Dissolved oxygen emerges as a principal environmental factor shaping the distribution of bacterial communities within these farming areas [46]. Examining microorganisms' metabolic potentials via the reconstruction of metagenomic assembled genomes uncovers redundancies in pathways such as nitrate reduction, taurine metabolism, 1-aminocyclopropane-1-carboxyl degradation, auxin, and vitamin B, alongside distinctive metabolic pathways, including ammonia oxidation, denitrification, and sulfide oxidation, as observed in specific taxa [47].

## 3. Effects of mariculture on marine sediment bacterial communities

Suspended marine aquaculture, which is a prevalent practice in open sea areas, involves cultivating aquatic organisms using structures such as cages and floating rafts. This leads to the deposition of uneaten feed and feces in sediments, significantly impacting the benthic ecosystem's stability [48]. Alterations in critical environmental factors, including dissolved oxygen, water content, NO^3−^, and particulate organic carbon, play a crucial role in shaping bacterial communities' distribution [48]. Consequently, alterations in these environmental conditions exert direct effects on the diversity and structural characteristics of bacterial communities [49].

Marine aquaculture disrupts sediment ecosystems, influencing bacterial communities by increasing the nutrient and heavy metal content. A study on submersible salmon farming cages in the Yellow Sea revealed a reduction of bacterial abundance in sediments due to aquaculture [50]. Similarly, research assessing the effects of intensive terrestrial fish farming in Qingdao on sedimentary environments indicated that excessive nutrients present during the cultivation process were likely to diminish the diversity of bacterial communities within the sediments [51]. Conversely, an analysis of bacterial community diversity in intertidal sediments of the Bohai Sea suggested that aquaculture pollution might enhance the sediment bacterial diversity [52]. In studies concerning the comprehensive cultivation of fish and shrimp, the predominant bacterial communities identified in the sediments were Proteobacteria and Bacteroidetes, displaying higher similarities and greater biodiversity levels in sediment bacterial samples compared to those of planktonic bacteria [15]. Different farming methods and areas showed varying impacts on sediment bacterial communities, with environmental fluctuations caused by marine aquaculture affecting the richness of these communities [53]. Bacteria in marine ecosystems degrade organic carbon in aquaculture sediments into small inorganic substances for use by other organisms, with an increased abundance of bacterial communities specializing in organic carbon degradation, such as Chloroflexi and Planctomycetes, as observed in marine aquaculture areas [54].

Heavy metal pollution poses a significant challenge in marine aquaculture, notably exacerbated using antifouling coatings on fishing nets and other equipment, which elevates heavy metal concentrations in sediments [55]. Furthermore, the deployment of contaminated or substandard feeds contributes to the accumulation of heavy metals in both cultured organisms and sediments, underscoring the critical impact of such pollution on microbial communities within these sediments [56]. Particularly, the heavy metals generated from aquaculture activities tend to accumulate in sediment layers, thus affecting the surrounding ecosystems, including mangroves, tidal flats, and aquaculture ponds. A study on heavily polluted mangroves in southern China revealed that heavy metals predominantly accumulated in surface sediments and significantly impacted the bacterial community [57]. Specifically, metals such as As, Co, Ni, and Pb showed a positive correlation with the presence of Bacteroidetes, *Actibacter*, and *Sphingobacterium*, while negatively correlated with the abundance of *Holophaga* and *Caldithrix* [57]. Certain bacteria, such as *Pseudomonas* and *Burkholderia*, showed potential for heavy metal bioremediation, thus indicating their role in heavy metal transport mechanisms [58]. However, research on heavy metal impacts on sediment bacterial communities in aquaculture settings is limited, highlighting the need for further investigation to understand these interactions fully. Unlike aquatic animals, seaweeds effectively remediate heavy metals, with *Gracilaria* (Rhodophyta) farming areas showing lower concentrations of Cd, Cu, Zn, and Pb in surface sediments compared to other farming regions [59]. Utilizing algae's advantages can modify farming practices, reduce pollution, and enhance environmental benefits. However, further exploration is needed to understand the role of seaweed farming in remediating sediment heavy metals for different seaweed species.

Examining the impact of mariculture on bacterial communities in sediments is crucial for mitigating harm to marine ecosystems from organic pollutants, heavy metals, and other contaminants. Sediments are integral to marine ecosystems, thus emphasizing the need to improve the management and surveillance of the sediment environment for effective protection. Preserving species diversity greatly promotes the sustainable development of marine aquaculture.

## 4. Effects of marine aquaculture on fungal and viral communities

Fungi and viruses are prevalent in the ocean, yet their role in mariculture remains understudied. As vital components of ecosystems, fungi respond to marine aquaculture and play a role in nutrient decomposition. In freshwater aquaculture settings, such as ponds and lakes, dominant fungi include Ascomycota, Chytridiomycota, Basidiomycota, and unclassified fungi. Variations in sediment environmental factors influence fungal communities in ponds dedicated to fish, crayfish, and crabs. Fishpond sediments exhibit a higher abundance of unclassified fungi, while Rozellomycota is less abundant than in crab and crayfish ponds [60]. Fungal community phylogenetic diversity positively correlates with the sediment pH, the total nitrogen, and the total phosphorus content [61]. Research on *Porphyra alba* cultivation underscores the seawater's pivotal role in eukaryotic biodiversity. Initially, Cryptophyta dominates the early growth stages, but as development progresses, groups such as Collector and Cryptophyta become more prominent [62]. Fungal diversity significantly increases in semi-enclosed bays, driven by mariculture-induced changes in the seawater temperature, dissolved oxygen, and nitrite levels [63]. Yet, eukaryotic microorganism distribution between water and sediment shows less variation than bacteria [64]. At present, our research on the impact of marine aquaculture on fungal communities is insufficient and further research is needed to verify it.

Marine viruses play a dual role in aquaculture, acting as beneficial bacteriophages impeding pathogenic bacteria and as pathogens causing illness in marine organisms. *Vibrio parahaemolyticus*, which is a prevalent pathogenic bacterium, poses a threat to both marine organisms and humans, thus resulting in illnesses. *V. parahaemolyticus* phages such as F23s2, H256D1, and the recently discovered phage phiTY18 showed inhibitory effects, thus offering alternatives to antibiotics to control *V. parahaemolyticus* [65],[66]. However, certain viruses, such as the white spot syndrome virus, the Singapore grouper iridovirus, and the infectious subcutaneous and hematopoietic necrosis viruses in aquaculture, exhibit a high fatality rate, thus posing a severe threat to crustaceans and groupers and hindering economic development [67]–[69].

Investigating the virus community diversity within sediments affected by various shrimp farming methods revealed Caudovirales as the predominant viral taxa [70]. Introducing different species in shrimp farming induced shifts in the composition and diversity of the viral community. Incorporating seaweed into shrimp ponds increased the relative abundance of Phycodnaviridae and Podoviridae, with a decrease in the relative abundance of Siphoviridae [70]. The seawater culture's impact on the sediment characteristics created an environment conducive to virus growth and reproduction, resulting in numerous viruses, including many unexplored variants [48]. Despite the recognized significance of understanding the repercussions of marine aquaculture on virus communities, the existing research in this domain remains limited, necessitating further scholarly inquiry and exploration.

Exploring the influence of marine aquaculture on fungal and viral communities provides insights into species diversity. This understanding can enhance disease prevention and diagnosis strategies in aquaculture, facilitate the selection of breeding methods tailored to aquaculture areas, and contribute to the advancement of aquaculture economics.

## 5. Effects of marine aquaculture on the distribution of ARGs

Marine aquaculture heavily relies on antibiotics for disease control, thus leading to the release of these substances into wastewater and contributing to the emergence of antibiotic-resistant bacteria [71]. Antibiotic overuse results in the widespread expression of ARGs, particularly in sediments, with Proteobacteria and Bacteroidetes identified as primary hosts. The horizontal transfer of ARGs affects bacterial communities in both marine and terrestrial environments, thus posing risks to marine life and human health as antibiotic-resistant bacteria proliferated in sediments. Coastal aquaculture farms exhibited higher antibiotic levels [72],[73]. Investigating the impact of distance from the sea on the ARGs abundance and diversity would be beneficial in understanding their impact on marine ecosystems.

In marine aquaculture, continuous feed supplementation is essential to meet the nutritional needs of aquatic organisms, thus leading to the sedimentation of uneaten feed and fecal matter and an increase in the nutrient and heavy metal concentrations. The use of fishmeal in aquaculture has been linked to an elevated abundance and diversity of ARGs in animals [74]. A sediment survey in the coastal fish farming areas of the Yellow River Delta revealed a significant positive correlation between the sediment concentrations of Cu and Cr and the abundance of specific ARGs [75]. The impact of marine aquaculture on bacterial community dynamics was observed in a survey of the grouper culture system in Hainan, China, indicating a positive correlation between Proteobacteria, Actinobacteria, Cyanobacteria, and various ARGs, thus suggesting their potential role as dominant hosts [76]. Perturbations introduced by marine aquaculture, including nutrients, heavy metals, and alterations in the bacterial communities, collectively influence the distribution of ARGs. Distinctions in the bacterial communities among diverse aquaculture systems emerge as the principal driving factors that influence the ARGs distribution [77].

The existing research on antibiotic resistance in marine aquaculture areas is incomplete. To effectively address the issue of the ARGs pollution stemming from aquaculture and comprehend the factors that influence the distribution of ARGs, additional research and monitoring are necessary. Implementing targeted management measures can help control the dissemination of ARGs in a timely manner.

## 6. The rise, advantages, and prospects of IMTA

IMTA represents a sustainable approach to marine aquaculture, characterized by the co-cultivation of species at different trophic levels within aquaculture farms. IMTA aims to mitigate the environmental impact of waste generated during the breeding process through nutrient circulation, thus establishing itself as a model for sustainable marine aquaculture.

Fish farming in coastal areas, particularly during Pacific salmon cultivation, contributes to seawater eutrophication, generating particulate matter and dissolved waste [78]. IMTA introduces organisms such as algae, shellfish, and sea cucumbers to utilize fish-generated waste, thus reducing biological pollution and harmful algal blooms [78]. The inclusion of *M. edulis*, seaweed, and other organisms in coastal fish cage farming enhances water and sediment quality, absorbs waste from fish farming, and serves as a nutrient source during breeding, thus contributing to increased economic benefits [79]. This integrated approach addresses environmental concerns while promoting sustainable practices for both ecological and economic advantages.

In Sanggou Bay [80], IMTA, specifically kelp-scallop integrated farming, is more sustainable than monoculture. Kelp absorbs organic and inorganic waste from shellfish, thus promoting nutrient circulation and reducing costs. It acts as a biological filter, controlling water eutrophication and enhancing marine ecosystem health. Small-scale co-cultivation of seaweed and shellfish in IMTA has a minimal impact on water nutrient levels and plankton mass [81]. Larger-scale seaweed farming may influence dissolved nutrient concentrations and plankton diversity. In the co-cultivation of kelp and *M. edulis*, IMTA increases the kelp biomass, reduces periphyton coverage, and enhances kelp growth through *M. edulis* excrement [82]. Model simulations confirm IMTA's bioremediation potential, thus reinforcing its environmental benefits [83].

In IMTA, Proteobacteria and Bacteroidetes dominate both water bodies and sediments, with seasonal variations in bacterioplankton and higher biodiversity in sediment bacteria [15]. These bacteria primarily function in amino acid transport and metabolism, peaking in sediment bacteria in January and in bacterioplankton during April [15]. In Ailian Bay's IMTA area, bacteria involved in sulfide recycling and organic consumption are abundant. Long-term mariculture significantly influences total bacterial communities, with minor effects in benthic bacterial communities [84]. In fishery ponds, sediment bacterial communities are characterized by Proteobacteria prevalence, followed by Actinobacteria, Bacteroidetes, Firmicutes, and Cyanobacteria, with tetracycline resistance genes commonly found [85]. Introducing mullet in shrimp ponds reduces the *Vibrio harveyi* concentration, thus decreasing the likelihood of shrimp disease [86]. Optimal mullet stocking density improves the water quality and biomass in shrimp-fish co-culture ponds, thus creating an ideal environment for bacterial growth [87]. The introduction of benthic suspension-feeding bivalves alters the pond IMTA, impacting sediment microbial communities. The dominant bacterial genus *Halomonas* increases from day 7 to day 14, declining to initial levels on the 35th day [88]. *Paenisporosarcina* peaks on the 7th day and gradually decreases thereafter. With *Mercenaria mercenaria* (Mollusca, Bivalvia) stocking, *Paenisporosarcina* becomes the second dominant bacterial genus, and increased *Ruditapes philippinarum* (Mollusca, Bivalvia) stocking reduces the *Gramella* and *Salinimicrobium* abundance [88]. IMTA induces bacterial community structure changes, with biotic and abiotic factors affecting the microbial compositions. In fish-algae co-culture, *Marinicella* and Saprospiraceae dominate among Bacteroidetes, while fish-shrimp-algae co-culture exhibits *Aureimarina* and *Robiginitalea* dominance [89]. Fish-shrimp co-culture shows dominance by Epsilonproteobacteria and Gammaproteobacteria (for example *Acinetobacter* and *Litoricola*). Flavobacteriia and Bacteroidia among Bacteroidetes, and Gammaproteobacteria and Betaproteobacteria within Proteobacteria, emerge as potential indicators [89].

However, the implementation of IMTA comes with potential risks, including the transfer of allergens by seaweed co-cultivated with fish, mollusks, and crustaceans [90]. Heavy metal pollution in aquaculture is a public health issue, particularly in the western Guangdong's fish and shrimp cultivation zones, where it significantly affects the Cu, Cr, and Pb levels [91]. Water pH and dissolved oxygen influence these metal concentrations, thus creating differences in Pb and Cu between aquaculture ponds and cages [91]. Economic challenges, such as rising labor costs and declining sales profits, also need consideration [92]. Despite these challenges, the scientifically grounded and ecologically advantageous nature of IMTA, especially animal-algae integrated farming, positions it as a promising approach for sustainable marine aquaculture.

## 7. Problems and prospects

Mariculture induces changes in critical water and sediment parameters, including dissolved oxygen, organic matter, nitrogen, phosphorus, and pollutants such as heavy metals and ARGs. These alterations have a profound impact on microbial communities. The breeding area exhibits distinct patterns of species enrichment and an altered aquatic environment, thus significantly deviating from uncultivated areas. Systematic monitoring of microbial communities offers valuable insights into the environmental implications of marine aquaculture, which are crucial to promptly address issues and ensure sustainable practices that align with the growing demand for seafood.

The growth of marine aquaculture introduces substantial environmental hurdles, particularly through pollution via direct wastewater discharge that degrades the coastal water quality. Effective marine agriculture management, embodied by practices such as IMTA, can bolster environmental sustainability and elevate economic performance. Such methodologies thrive with governmental backing and the judicious selection of species that are well-suited to local ecosystems, which are essential to foster sustainable development. Moreover, harnessing biotechnology unveils promising avenues for resolution. Specifically, microbial communities serve as bioindicators to track persistent organic pollutants in marine sediments, thus playing a pivotal role in efficient pollution management in aquaculture wastewater systems and safeguarding the marine environment [93]. Through biofilm formation or the production of extracellular polymers, isolated marine microorganisms are pivotal in pollutant remediation and improving the water quality [94]. Thus, these microorganisms are indispensable in both detecting and mitigating pollution, thus underscoring the multifaceted approach needed to ensure the sustainability of marine aquaculture.

This review aims to provide a comprehensive understanding of the impact of mariculture on microbial communities. While numerous studies have investigated changes in bacterial communities during mariculture, limited attention has been directed toward marine microorganisms, including unclassified fungi and virus communities. Understanding the broader effects of mariculture on the marine ecosystem requires exploration into the impact on various fungal and virus communities' composition, diversity, metabolic functions, and distribution patterns. Additionally, the influence of marine aquaculture on the distribution of emerging contaminants, such as ARGs in the ocean, remains poorly understood. Studying changes in microbial communities can provide an in-depth understanding of the impact of aquaculture on marine ecosystems, which is beneficial to promoting marine environmental protection and to achieve sustainable development of marine aquaculture.
